# In Vitro Study of Cyano-Phycocyanin Release from Hydrogels and Ex Vivo Study of Skin Penetration

**DOI:** 10.3390/ph17091224

**Published:** 2024-09-17

**Authors:** Daiva Galinytė, Jurga Bernatoniene, Modestas Žilius, Kristina Rysevaitė-Kyguolienė, Arūnas Savickas, Jūratė Karosienė, Vitalis Briedis, Dainius Haroldas Pauža, Nijolė Savickienė

**Affiliations:** 1 Department of Pharmacognosy, Faculty of Pharmacy, Academy of Medicine, Lithuanian University of Health Sciences, Sukilėlių av. 13, 50162 Kaunas, Lithuania; nijole.savickiene@lsmu.lt; 2 Department of Drug Technology and Social Pharmacy, Faculty of Pharmacy, Academy of Medicine, Lithuanian University of Health Sciences, Sukilėlių av. 13, 50162 Kaunas, Lithuania; jurga.bernatoniene@lsmu.lt (J.B.); arunas.savickas@lsmu.lt (A.S.); 3 Department of Clinical Pharmacy, Faculty of Pharmacy, Academy of Medicine, Lithuanian University of Health Sciences, Sukilėlių av. 13, 50162 Kaunas, Lithuania; modestas.zilius@lsmu.lt (M.Ž.); vitalis.briedis@lsmu.lt (V.B.); 4 Institute of Anatomy, Faculty of Medicine, Academy of Medicine, Lithuanian University of Health Sciences, Mickevičiaus St. 9, 44307 Kaunas, Lithuania; kristina.rysevaite@lsmu.lt (K.R.-K.); dainius.pauza@lsmu.lt (D.H.P.); 5 Laboratory of Algology and Microbial Ecology, Nature Research Centre, Akademijos St. 2, 08412 Vilnius, Lithuania; jurate.karosiene@gamtc.lt

**Keywords:** cyano-phycocyanin, hydrogel, sodium alginate, propylene glycol, in vitro drug release, ex vivo skin penetration

## Abstract

Background: This study explored the most suitable materials for incorporating cyano-phycocyanin (C-PC) into hydrogels, focusing on maintaining the C-PC’s long-term structural integrity and stabilityNext, the release of C-PC from the hydrogels and its skin penetration were investigated. Methods: A series of 1% (*w*/*w*) C-PC hydrogels was prepared using various gelling agents and preservatives. Spectrophotometric measurements compared the amount of C-PC in the hydrogels to the initially added amount. After selecting the most suitable gelling agent and preservative, two C-PC hydrogels, with and without propylene glycol (PG) (Sigma-Aldrich, St. Louis, MO, USA), were produced for further testing. In vitro release studies utilized modified Franz-type diffusion cells, while ex vivo skin-permeation studies employed Bronaugh-type cells and human skin. Confocal laser scanning microscopy analyzed C-PC accumulation in the skin. Results: The findings demonstrated that sodium alginate (Sigma-Aldrich, St. Louis, MO, USA), hydroxyethyl cellulose (HEC) (Sigma-Aldrich, St. Louis, MO, USA), and Soligel^TM^ (Givaudan, Vernier, Switzerland) are effective biopolymers for formulating hydrogels while maintaining C-PC stability. After 6 h, C-PC release from the hydrogel containing PG was approximately 10% or 728.07 (±19.35) μg/cm^2^, significantly higher than the nearly 7% or 531.44 (±26.81) μg/cm^2^ release from the hydrogel without PG (*p* < 0.05). The ex vivo qualitative skin-permeation study indicated that PG enhances C-PC penetration into the outermost skin layer. Conclusion: PG’s ability to enhance the release of C-PC from the hydrogel, coupled with its capacity to modify the skin barrier ex vivo, facilitates the penetration of C-PC into the stratum corneum.

## 1. Introduction

C-PC is a supramolecular protein–chromophore complex present in all cyanobacterial species [1,2]. Recent studies have increasingly highlighted C-PC’s potential for treating various skin conditions due to its ability to promote tissue regeneration, enhance wound healing, and exhibit antioxidant, antimicrobial, and anti-inflammatory properties [3]. For instance, in vivo wound-healing research confirmed that incorporating C-PC into hydrogels enhances the antioxidant capacity, reduces the levels of inflammatory markers (IL-6, IL-1β, TNF-α), and accelerates the wound-healing process [4]. Additionally, C-PC demonstrated significant anti-photoaging effects in UVB-induced mouse skin by preventing collagen-fiber degradation, enhancing antioxidant enzyme activity, and reducing inflammatory factor expression [5]. Subcutaneous injections of C-PC in melanoma-induced mice increased the absolute number of T lymphocytes and myeloid cells, showing a positive immunomodulatory effect and reducing tumor tissue [6]. Both in vitro and in vivo studies suggested that C-PC can increase the activation of pro-apoptotic genes and suppress anti-apoptotic genes, indicating its potential as a treatment for skin cancer [3].

C-PC applications are dependent upon its purity, measured by the absorbance ratio A620/A280, where A620 is the absorbance of C-PC at 620 nm, and A280 is the absorbance of other proteins at 280 nm [7]. C-PC with a purity greater than 0.7 is suitable for food, greater than 1.5 for cosmetics, and greater than 4.0 for therapeutic treatments [8]. However, the unique structure of C-PC presents challenges to developing effective topical formulations, as various factors, such as temperature, pH, and excipients, can affect its stability during manufacturing [9,10]. The C-PC consists of a heterodimeric monomer composed of α and β subunits, where each α subunit is bound to one bilin chromophore and each β subunit is associated with two bilin chromophores. These monomers formed a ring-shaped trimer (αβ)_3_, which further assembles into hexameric structures [(αβ)_3_]_2_ [11,12]. Phycocyanobilins are responsible for C-PC’s distinctive blue color. In vitro, C-PC solutions usually contain various oligomeric states, such as trimers, hexamers, and occasionally, monomers, with molecular weights ranging from approximately 44 to 260 kDa [1,13]. Compounds that interact with the tetrapyrrole structure, such as oxygen, free radicals, and acids, can accelerate C-PC degradation. Alterations in the chromophore’s structure can fade color and reduce therapeutic activity. [12]. Therefore, the preservation of C-PC’s structural stability is essential when developing topical applications with this promising natural material.

Hydrogels, which are three-dimensional polymeric materials with high water content, are an excellent medium for incorporating hydrophilic substances like C-PC [14,15]. Suitable gelling agents that do not compromise C-PC stability are critical in these formulations. Synthetic polymers such as crosslinked polyacrylic acid, commercially available as Carbopol^®^ (The Lubrizol Corporation, Wickliffe, OH, USA) and Pemulen^TM^ (The Lubrizol Corporation, Wickliffe, OH, USA) are commonly used due to their properties, including viscosity, transparency, and bioadhesion [16,17,18,19]. Poloxamer 407 (Sigma-Aldrich, St. Louis, MO, USA), a copolymer of ethylene oxide and propylene oxide, can also be used to create thermosensitive hydrogels that are suitable for localized therapy and enhanced drug permeation. [20,21,22]. Biopolymers, like sodium alginate, cellulose, and chitosan, are valued for their biocompatibility, biodegradability, and environmental sustainability [23]. Sodium alginate, obtained from brown seaweeds or microbial fermentation, is composed of β-d-mannuronic acid and α-l-guluronic acid units [24]. Its characteristics, including rapid gelation, outstanding biocompatibility, and low cost, vary depending on the algal species from which it is derived. [23,25,26,27]. Cellulose is a natural polymer composed of glucose monomers, forming the fundamental structural component in plant cell walls, and is found in fibers like flax and cotton. It can also be of bacterial origin (e.g., Acetobacter xylinum). Water-soluble cellulose derivatives are typically produced by etherifying cellulose, reacting its hydroxyl groups with organic compounds such as methyl and ethyl groups [28]. Chitosan, a biopolymer obtained from the skeletal structures of crustaceans, insect cuticles, and fungal cell walls, is composed of copolymers of glucosamine and N-acetylglucosamine. Chitosan enhances epithelialization and exhibits both wound healing and antimicrobial properties [15,23,29]. However, its application is limited by its low solubility in neutral and basic pH conditions, so it is frequently employed in the form of hydrogels prepared by dissolving chitosan in an acidic solution [29,30]. Soligel^TM^, a biopolymer produced biotechnologically using Rhizobium sp., is a branched polysaccharide consisting of a sequence of monomer units that includes six neutral sugars (three glucose and three galactose molecules) in the pyranose form, along with one glucuronic acid and a pyruvyl substituent [31]. 

After applying the hydrogel to the skin, the drug must first be released from its vehicle and then partitioned into the outermost layer of the skin, the stratum corneum, which serves as the primary rate-limiting step for its delivery. For pharmaceuticals, the skin presents both a challenge as a barrier and an opportunity due to its large surface area for drug delivery [32,33]. For passive absorption and permeation through the stratum corneum, the substance must possess specific physicochemical characteristics, such as a molecular weight of less than 500 kDa, moderate lipophilicity, and suitable solubility in both lipids and water [34]. Thus, another challenge for topical formulations with C-PC is high molecular weight and hydrophilicity. Enhancing the skin permeation of drugs is a crucial focus in pharmaceutical research [35,36]. PG, a chemical penetration enhancer commonly used in topical formulations, can overcome these barrier properties and facilitate drug transport across the skin by disrupting the diffusional pathways within the skin, thereby increasing drug permeability [37,38] However, there have been no studies on the ability of PG to promote the skin penetration of C-PC.

In pursuit of a versatile biomaterial platform, this investigation explored the most suitable material compositions for fabricating a hydrogel that could integrate C-PC, with a steadfast emphasis on ensuring its structural integrity and stability. Additionally, the research sought to evaluate the release of C-PC from the formulated hydrogel and assess the extent of C-PC penetration into the skin.

## 2. Results

### 2.1. Selection of Gelling Agent

**Figure 1:**
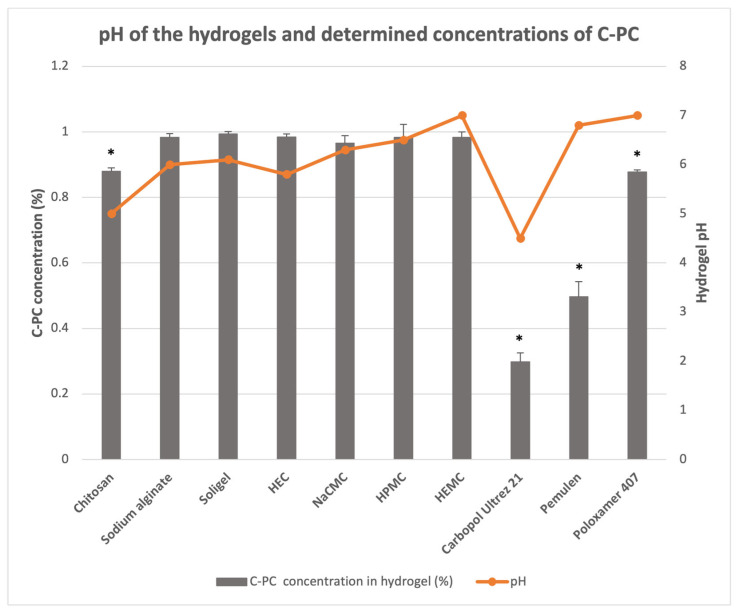
pH of hydrogels and determined concentrations of C-PC in the produced hydrogels with different gelling agents. An asterisk (*) indicates statistically significant differences between the added and determined concentrations of C-PC (*p* < 0.05), evaluated using a paired-sample *t*-test. The means and standard deviations are presented. The experiment was repeated three times.

In this study, C-PC was isolated from cyanobacterial biomass collected from the Kaunas Lagoon (Kaunas, Lithuania), with a purity of 1.5 (0.006). To identify the most suitable gelling agent, the research team prepared a series of 1% (*w*/*w*) C-PC hydrogels using a variety of polymeric materials as the primary structural components. The gelling agents and their concentrations were selected for this study based on information from the literature sources and technical data sheets provided by the manufacturers. The study focused on three synthetic polymers at the following concentrations: Carbopol^®^ Ultrez 21 (Lubrizol Corporation, Wickliffe, OH, USA)- 0.8% (*w*/*w*) [18,29], Pemulen^TM^ (Lubrizol Corporation, Wickliffe, OH, USA)—0.5% (*w*/*w*) [19], and Poloxamer 407 (Sigma-Aldrich, St. Louis, MO, USA)—15% (*w*/*w*) [22]. Additionally, seven biopolymers were studied, including sodium alginate (Sigma-Aldrich, St. Louis, MO, USA)—3.5% (*w*/*w*) [39,40], hydroxyethyl cellulose (HEC) (Sigma-Aldrich, St. Louis, MO, USA)—5.5% (*w*/*w*) [41], sodium carboxymethyl cellulose (NaCMC) (Sigma-Aldrich, St. Louis, MO, USA)—5% (*w*/*w*) [42], hydroxy-propyl methylcellulose (HPMC) (Sigma-Aldrich, St. Louis, MO, USA)—15% (*w*/*w*) [41,43], hydroxyethyl methylcellulose (HEMC) (Sigma-Aldrich, St. Louis, MO, USA)—3% (*w*/*w*) [44], chitosan 80/1000 molecular weight (chitosan) (Sigma-Aldrich, St. Louis, MO, USA)—3% (*w*/*w*) [29], and Soligel^TM^ (Givaudan, Vernier, Switzerland)—1.5% (*w*/*w*) [31]. The stabilities of the C-PC in these hydrogels were evaluated spectrophotometrically by measuring the C-PC content in the prepared hydrogels and comparing it with the initial amount of C-PC added. The hydrogels were stored at +4 °C for 48 h before analysis. As depicted in Figure 1, the results indicated that C-PC remained structurally stable in hydrogels containing sodium alginate, Soligel^TM^, HEC, NaCMC, or HEMC, with no statistically significant decrease in C-PC concentration observed (*p* > 0.05). However, in the formulations with Poloxamer 407 and chitosan, the C-PC content decreased by 12.20% and 12.00%, respectively (*p* < 0.05). The most significant instability was observed in hydrogels with Carbopol^®^ Ultrez 21, where the C-PC content was 71.80% lower than the initial amount (*p* < 0.05), and with Pemulen^TM^, which showed a decrease of 50.30% (*p* < 0.05). A visible fading of the hydrogels’ color accompanied the decrease in C-PC concentration. 

Based on these findings, six polysaccharide-based biopolymers—sodium alginate, Soligel^TM^, HEC, NaCMC, HPMC, and HEMC—were selected for further research, as no statistically significant changes in C-PC concentration were observed with these gelling agents.

### 2.2. Selection of Preservative for C-PC Hydrogels 

**Figure 2:**
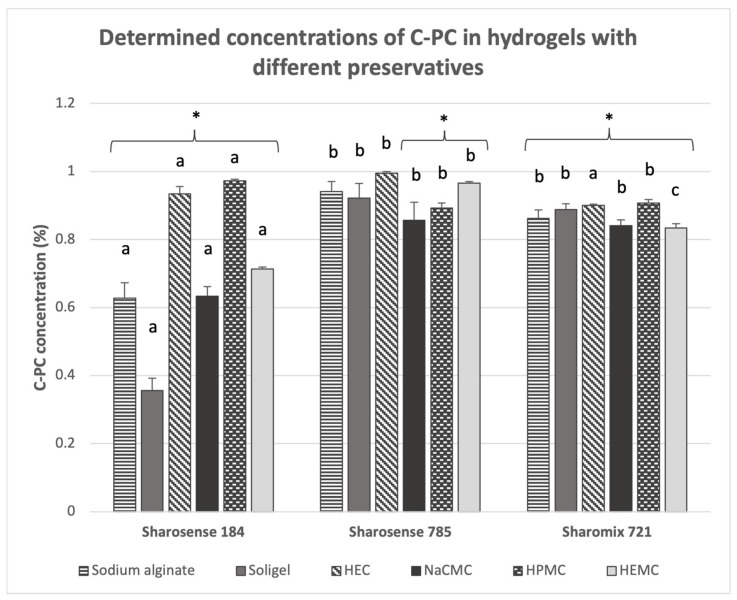
Determined concentrations of C-PCs in hydrogels with different preservatives. Different letters denote statistically significant differences (*p* < 0.05) in the C-PC concentrations among hydrogels containing the same gelling agent but different preservatives, as assessed using Bonferroni’s test. An asterisk (*) indicates statistically significant differences (*p* < 0.05) between the initially added and the determined C-PC concentrations, evaluated using a paired-sample *t*-test. The means and standard deviations are presented. The experiment was repeated three times.

The next phase of the study involved screening to identify the most suitable preservative system for the C-PC-containing hydrogels. Eighteen C-PC hydrogels were prepared using in the biopolymers selected in the previous stage—sodium alginate, Soligel™, HEC, NaCMC, HPMC, and HEMC—and three environmentally friendly preservatives, namely SharoSENSE™ Plus 184 (SHARON Laboratories, Ashdod, Israel) (maltol, didecyldimonium chloride), SharoSENSE™ Plus 785 (SHARON Laboratories, Ashdod, Israel) (maltol, sorbic acid, sodium benzoate), and Sharomix™ 721 (SHARON Laboratories, Ashdod, Israel) (dehydroacetic acid, benzyl alcohol, water). The hydrogels were stored at +4 °C for 48 h before undergoing spectrophotometric analysis. The stability of the C-PC in these hydrogels was assessed by measuring the C-PC content spectrophotometrically and comparing it with the initial amount of C-PC added during the formulation. The changes in C-PC concentrations across the hydrogels containing different preservative systems are illustrated in Figure 2. 

When SharoSENSE Plus^TM^ 184 was used with gelling agents, such as sodium alginate, Soligel ^TM^, HEC, NaCMC, and HEMC, the C-PC concentration was significantly lower (*p* < 0.05) compared to hydrogels containing SharoSENSE^TM^ Plus 785 and Sharomix^TM^ 721. Depending on the gelling agent, the C-PC concentration decreased by 6.60% to 64.40% in combination with SharoSENSE Plus^TM^ 184, with visual color changes observed in the hydrogels. In contrast, the combination of SharoSENSE Plus^TM^ 184 with HPMC resulted in a higher C-PC concentration than the other hydrogels containing HPMC but different preservative systems (*p* < 0.05). Hydrogels containing SharoSENSE^TM^ Plus 785 and Sharomix^TM^ 721 showed smaller reductions in their C-PC concentration compared to those with SharoSENSE PlusTM 184. Specifically, Sharomix^TM^ 721 resulted in a C-PC concentration decrease of 9.30% to 16.60%, while SharoSENSE^TM^ Plus 785 caused a reduction of 0.5% to 14.30%, depending on the specific gelling agent used. The smallest reductions in C-PC concentration were observed with SharoSENSE^TM^ Plus 785 compared to the other preservative systems. A statistically significant difference (*p* < 0.05) in C-PC reduction was found when using SharoSENSE Plus^TM^ 184 and Sharomix^TM^ 721 with all tested gelling agents. However, no statistically significant difference (*p* > 0.05) was observed with SharoSENSE^TM^ Plus 785 in combination with sodium alginate, Soligel^TM^, or HEC.

Therefore, based on the structural stability of C-PC in hydrogels with different preservatives, the combinations of sodium alginate, Soligel^TM^, and HEC with SharoSENSE^TM^ Plus 785 were selected for further investigation. 

### 2.3. Microbiological Evaluation of C-PC Hydrogels

Three different C-PC hydrogels were formulated for microbiological analysis using selected gelling agents, namely sodium alginate, Soligel^TM^, and HEC, combined with the preservative SharoSENSE^TM^ Plus 785 (maltol, sorbic acid, and sodium benzoate). The formulations were as follows:

- I C-PC gel: C-PC (1% *w*/*w*), glycerol (Sigma-Aldrich, St. Louis, MO, USA) (5% *w*/*w*), sodium alginate (3.5% *w*/*w*), and Sharosense 785 (0.05% *w*/*w*);
- II C-PC gel: C-PC (1% *w*/*w*), glycerol (5% *w*/*w*), Soligel (1.5% *w*/*w*), and Sharosense 785 (0.05% *w*/*w*);
- III C-PC gel: C-PC (1% *w*/*w*), glycerol (5% *w*/*w*), HEC (5.5% *w*/*w*), and Sharosense 785 (0.05% *w*/*w*).

Glycerol did not significantly impact the stability of C-PC in the hydrogels.

The microbiological evaluation of C-PC hydrogels was performed at the Kaunas Department of the National Public Health Care Laboratory (Kaunas, Lithuania). 

The results, presented in Table 1, indicated that the pathogens *Staphylococcus aureus*, *Pseudomonas aeruginosa*, *Candida albicans,* and *Escherichia coli* were not detected in the C-PC hydrogels. The count of aerobic mesophilic bacteria was less than 1.0 × 10^1^ CFU/g. These findings confirm the excellent antimicrobial properties of SharoSENSE^TM^ Plus 785 in all tested C-PC hydrogels. 

### 2.4. In Vitro Evaluation of C-PC Release from Experimental Hydrogels

Based on the structural stability of C-PC and the microbiological evaluation of the C-PC hydrogels, the combinations of sodium alginate, Soligel™, and HEC with SharoSENSE™ Plus 785 were identified as suitable formulations for C-PC hydrogels development. Among these, sodium alginate was selected for further investigation as the gelling agent due to its shared origin with C-PC, with both being derived from algae. For the in vitro release study, two semisolid formulations were prepared, with their composition and pH shown in Table 2. The objective of this study was to assess the influence of PG on the release of C-PC from the hydrogel matrix. The C-PC hydrogel without PG served as the control.

**Figure 3:**
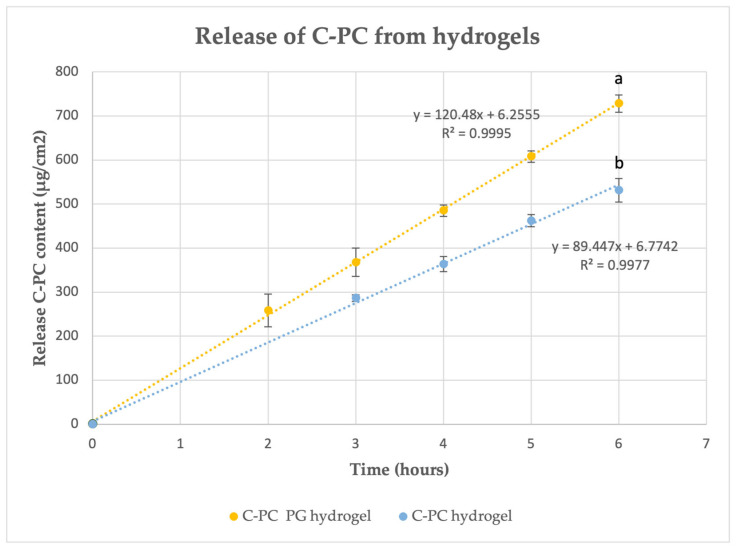
The release of C-PC from hydrogels: C-PC PG hydrogel and C-PC hydrogel without PG (control). Different letters indicate statistically significant differences (*p* < 0.05) determined by independent-samples *t*-test. Means and standard deviations are presented. The experiment was repeated 3 times.

The release of C-PC from the PG-containing hydrogel was detected after 30 min and became quantitatively significant at 2 h, with 3.25% (258.304 (36.959) μg/cm^2^) of C-PC released. In comparison, the C-PC release from the hydrogel without PG was observed at 30 min but was quantitatively significant only after 3 h, with 3.59% (286.490 (7259) μg/cm^2^) of C-PC released (Figure 3). The determination coefficient ($R^{2}=0.9995)$ indicates an excellent fit of the linear model to the experimental data, suggesting a nearly perfect linear release of C-PC over time under the tested conditions. The slope values of the equations (120.48 for the PG hydrogel and 89.447 for the hydrogel without PG) represent the rate of C-PC release from the hydrogels per unit of time, with the higher slope in the PH hydrogel indicating a faster release compared to the control. After 6 h, the release data showed a statistically significant difference (*p* < 0.05) between the two formulations. The C-PC gel with PG released nearly 10% of the C-PC, amounting to 728.07 ± 19.35 µg/cm^2^, whereas the hydrogel without PG released approximately 7%, or 531.44 ± 26.81 µg/cm^2^. 

These findings indicate that the presence of PG significantly enhances the release rate of C-PC from the hydrogel, suggesting that the PG-containing formulation may be more suitable for applications requiring a quicker C-PC delivery. Conversely, the hydrogel without PG provides a more controlled, slower release, which could be advantageous for sustained-release applications. Further detailed studies are necessary to elucidate the exact mechanism by which PG facilitates C-PC release.

### 2.5. Ex Vivo Qualitative Assessment of C-PC Skin Permeation from Experimental Hydrogels 

In this pilot study, we qualitatively evaluated the influence of the penetration enhancer PG on the penetration of the high molecular weight hydrophilic protein C-PC into the skin. Two semisolid C-PC formulations, one containing PG and one without, were utilized for this investigation (Table 2).

**Figure 4:**
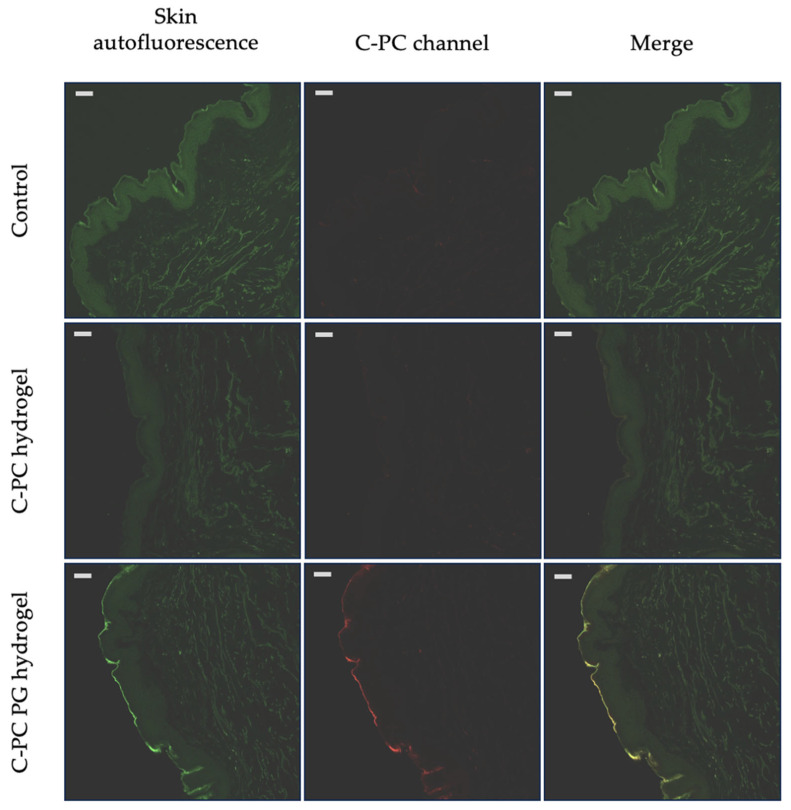
Confocal images of human skin autofluorescence (control) and human skin section after treatment with C-PC hydrogel and C-PC PG hydrogel. Scale bar 50 μm. The experiment was repeated 3 times.

The results of the ex vivo qualitative skin-penetration study are presented in Figure 4. The skin that was not treated with any hydrogel formulation served as the control (Figure 4, row Control). The fluorescent properties of C-PC were employed for qualitative assessment, with skin autofluorescence being excluded from the analysis (Figure 4, column C-PC channel). 

In the C-PC hydrogel row, significant skin autofluorescence is observed (Figure 4, column skin autofluorescence), but no fluorescence is detected in the C-PC channel, indicating that the C-PC did not penetrate the skin. This observation is consistent with the notion that C-PC, due to its high molecular weight and hydrophilic nature, does not penetrate the outermost layer of the skin—the stratum corneum—which is composed predominantly of ceramides, cholesterol, and fatty acids that contribute to its hydrophobic properties [45]. Conversely, the C-PC PG hydrogel formulation exhibited visible fluorescence in the C-PC channel (red color), indicating the presence of C-PC within the stratum corneum. 

Thus, the ex vivo skin-penetration study demonstrated that, within 24 h, C-PC was not detected in skin samples treated with the C-PC hydrogel lacking PG. In contrast, the C-PC from the C-PC PG hydrogel was predominantly localized in the stratum corneum, suggesting that the penetration-enhancer PG facilitates the penetration of C-PC into the skin.

## 3. Discussion

### 3.1. The Influence of Gelling Agents and Hydrogel Production Technology on the C-PC Stability

In this study, we aimed to identify the most suitable gelling agents for the production of C-PC hydrogel, with a focus on maintaining C-PC stability. Our investigation into C-PC stability within semi-solid formulations revealed that C-PC remained structurally stable in most formulations containing polysaccharide-based biopolymers, including sodium alginate, HEC, NaCMC, HPMC, HEMC, and Soligel^TM^. Studies conducted by other researchers have suggested that the incorporation of polysaccharides can effectively enhance C-PC stability. A common approach to improve protein stability is through glycation, a process wherein covalent cross-links form between the carbonyl groups of reducing sugars and the amino groups of the protein. The reaction can optimize the physicochemical properties of protein–sugar conjugates. For example, the glycation of C-PC has been shown to enhance its functionality. Polysaccharides with negatively charged functional groups, such as carboxyl or sulfate groups, can engage in electrostatic interactions with the positively charged amino acid residues on the surfaces of C-PC proteins, thereby improving the overall stability and functionality of the C-PC molecules [2]. However, a decrease in C-PC concentration was observed in the hydrogel containing chitosan. The heating of the chitosan hydrogel during manufacturing may have affected C-PC stability, despite careful monitoring to ensure the temperature did not exceed 40 °C. Previous research has indicated that C-PC stability significantly declines when the temperature surpasses the critical range of 45–47 °C, with the degradation rates increasing rapidly at higher temperatures [2,46]. These degradation reactions are likely associated with changes in the C-PC’s secondary, tertiary, and quaternary structures due to elevated temperatures [12]. Heat treatment has been shown to induce aggregation of C-PC subunits, contributing to its degradation and a noticeable reduction in the intensity of its characteristic blue coloration [2,46]. Additionally, a visible color fade was observed in hydrogels containing Carbopol^®^ Ultrez 21, Pemulen^TM^, and Poloxamer 407. The use of acetic acid in chitosan hydrogels, sodium hydroxide in Carbopol^®^ Ultrez 21 formulations, and triethanolamine in Pemulen^TM^ formulations may contribute to a reduction in C-PC stability. The pH values of the C-PC hydrogels containing chitosan, Poloxamer 407, Carbopol^®^ Ultrez 21, and Pemulen^TM^ were measured at 5.0, 7.0, 4.5, and 6.8, respectively. According to previous studies, C-PC exhibits optimal stability within a pH range of 5.5 to 6.0 [47]. These findings indicate that C-PC is sensitive to variations in both temperature and pH, and it is crucial to carefully monitor and maintain these parameters within optimal limits when producing formulations with C-PC.

### 3.2. Identifying an Efficient Preservative for C-PC Hydrogels 

Preservatives are a diverse group of chemical compounds commonly incorporated into cosmetic and pharmaceutical formulations to prevent microbial contamination and ensure product integrity. Despite their antimicrobial benefits, preservatives in skincare or treatment products can elicit adverse reactions, such as skin irritation or contact dermatitis [48]. The primary objective of this study was to identify an effective preservative system that is clean, safe, non-toxic, possesses a long shelf-life, and does not adversely impact the stability of C-PC. Three environmentally friendly preservatives were evaluated, namely SharoSENSE™ Plus 184 (maltol, didecyldimonium chloride), SharoSENSE™ Plus 785 (maltol, sorbic acid, sodium benzoate), and Sharomix™ 721 (dehydroacetic acid, benzyl alcohol, and water). SharoSENSE^TM^ Plus 785 was selected for further use in preserving C-PC hydrogels due to its minimal impact on C-PC stability.

The chosen preservative system, SharoSENSE^TM^ Plus 785, comprises three active ingredients, namely maltol, sorbic acid, and sodium benzoate. Benzoic acid (C_7_H_6_O_2_) is an aromatic carboxylic acid characterized by a carboxyl group (-COOH) directly attached to a benzene ring. It occurs naturally in various plant species, including fruits, vegetables, and fungi, with its concentration influenced by factors such as plant species, growing conditions, and environmental interactions [49]. Industrially synthesized benzoic acid and its derivates are commonly used as antibacterial and antifungal agents, as well as flavoring agents in food, cosmetics, and pharmaceuticals. While benzoic acid and its salts are generally considered safe within established limits, human exposure may occasionally exceed regulatory standards, and various absorption routes may be involved [50]. The preservative composition also includes sorbic acid, another organic acid with a broad-spectrum antimicrobial activity. Sorbic acid and sodium benzoate operate through similar mechanisms, inhibiting microorganisms by disrupting nutrient uptake, particularly by affecting the proton motive force (PMF) across microbial cell membranes. This disruption impairs the cell’s ability to maintain pH balance and transport essential molecules, leading to inhibited growth or cell death. Both sorbate and benzoate are particularly effective at low pH levels, with sorbic acid demonstrating greater efficacy between pH 4.0 and 6.0 compared to sodium benzoate [51]. Maltol, known as 3-hydroxy-2-methyl-4-pyrone, is a naturally occurring compound found in various plant sources, including *Larix deciduas*, *Evodiopanax innovans*, *Cercidiphyllum japonicum*, and *Panax ginseng*. It can also be produced by certain bacteria and molds. Maltol is widely used in the food industry as a flavoring agent due to its sweet aroma and is also used as a food additive and preservative. Although maltol’s antimicrobial activity is considered limited, requiring high concentrations (around 4000 ppm) to effectively inhibit microbial growth, it is regarded as non-toxic and generally recognized as safe (GRAS) for use in food and other consumer products [52]. 

Therefore, the SharoSENSE^TM^ Plus 785 preservative system serves as an effective bridge between natural and synthetic substances. The combination of its components may enhance the antimicrobial spectrum and potentially reduce the quantity of preservatives required, thereby minimizing the risk of adverse reactions in consumers.

### 3.3. Contextualizing the Microbiological Results of C-PC Hydrogels

To ensure the microbial safety of cosmetics and other topical products, it is essential to identify skin pathogens such as *Staphylococcus aureus*, *Pseudomonas aeruginosa*, and *Candida albicans*, as these can cause skin or eye infections. Additionally, detecting other microorganisms, including fecal contamination indicators like *Escherichia coli*, is crucial for verifying hygienic handling during the manufacturing process and ensuring high-quality production [53]. According to the European Standard EN ISO 17516:2014 [53], cosmetic products must be free from *Staphylococcus aureus*, *Pseudomonas aeruginosa*, and *Candida albicans*. The total count of aerobic mesophilic microorganisms should not exceed 1 × 10^2^ CFU per gram or milliliter for products intended for children under three years of age, for use around the eyes, or for application to mucous membranes, and should not exceed 1 × 10^3^ CFU per gram or milliliter for other cosmetic products [53]. 

### 3.4. Analysis and Implications of C-PC Release Profiles in Experimental Hydrogels

Topical formulations face additional challenges, with the critical factor being the release of the active substances before their penetration through the stratum corneum to exert a local or systemic effect [54]. In vitro studies involving the release of active substances through semipermeable synthetic membranes are conducted to evaluate the release process from semisolid formulations. The release of a drug before it penetrates the skin is a crucial consideration. Studies indicate that utilizing solvents to enhance drug release from the delivery system can significantly boost the drug’s skin-penetration capabilities, primarily due to the establishment of a higher concentration gradient [54]. Our in vitro C-PC-release study revealed that PG significantly promoted the faster release of C-PC from the hydrogel (*p* < 0.05). Several studies by other authors have also demonstrated the ability of PG to increase drug release from the formulation. Yang D. and other studies showed that PG significantly enhanced the release percent of Loxoprofen from polymer matrices consisting of an acrylic pressure-sensitive adhesive (PSA) containing a carboxyl group [55]. Wang Z. and others revealed that PG significantly increased the release of licochalcone A (a flavonoid compound extracted from the roots of *Glycyrrhiza glabra* L.) from a Carbomer 940 hydrogel by occupying the licochalcone A and Carbomer 940 binding site [56]. Thus, PG plays a crucial role in enhancing the release of active ingredients, such as C-PC, in in vitro experiments through several mechanisms. First, PG functions as a co-solvent, improving the solubility of both hydrophilic and lipophilic compounds within the hydrogel matrix. This increased solubility is critical because it can enhance the thermodynamic activity of the active ingredient, thereby driving its diffusion from the hydrogel into the surrounding medium [57]. In the specific context of this study, the inclusion of PG in the sodium alginate hydrogel formulation resulted in a statistically significant acceleration of C-PC release compared to the hydrogel without PG. This can be explained by PG’s ability to disrupt intermolecular interactions within the hydrogel matrix. Sodium alginate, a polysaccharide, forms a gel network that can effectively trap active ingredients like C-PC. However, PG is capable of weakening these interactions—possibly by interfering with hydrogen bonding or by occupying the binding sites between the C-PC molecules and the alginate chains. This disruption reduces the energy barrier for the diffusion of C-PC molecules, facilitating their release from the hydrogel [58]. Furthermore, PG may also influence the structural dynamics of the hydrogel matrix itself. It can cause partial swelling or plasticization of the polymer network, which increases the pore size within the gel, thereby creating additional pathways for the diffusion of the active ingredient [58]. The observed increase in the rate of C-PC release in the presence of PG aligns with previous findings in pharmaceutical research, where PG has been shown to enhance the release profiles of various drugs from polymeric carriers by modifying both the drug–polymer interactions and the physical structure of the carrier [58]. Moreover, sodium alginate is a gelling agent with a controlled release function [27]. The study on the release of C-PC from polylactic acid/phycocyanin–alginate composite cosmetic patches showed that the release of C-PC also depends on the ratio of C-PC to alginate. As this ratio decreases, the ability to release C-PC also decreases [59]. In our study, the concentration of C-PC in the gel was only 1%, so it is likely that, by increasing the concentration of C-PC and the ratio of C-PC to alginate, it would be possible to achieve a higher percentage of released C-PC.

Thus, the multifaceted action of PG not only promotes faster release but also contributes to a more consistent and predictable release profile, which is essential for achieving the desired therapeutic outcomes in topical drug delivery systems. Overall, the inclusion of PG in the hydrogel formulation is a key factor that modulates the release kinetics of C-PC, making it a critical component in the design of effective topical formulations.

### 3.5. Insights into Ex Vivo C-PC Skin Permeation from Experimental Hydrogels 

Cosmetic or pharmaceutical products applied topically face significant challenges in delivering active components into the deeper layers of the skin, as the stratum corneum, the outermost layer, serves as a highly effective barrier against the permeation of most compounds [60]. Our ex vivo qualitative skin-penetration study confirmed that high molecular weight and hydrophilic molecules, such as C-PC, have limited penetration ability into the stratum corneum. However, the presence of PG enhanced the penetration of C-PC into this outer layer. The correlation between the in vitro release of C-PC facilitated by PG and its subsequent ex vivo permeation through the stratum corneum warrants careful consideration. PG is well-established as a penetration enhancer, primarily due to its ability to modify both the physicochemical properties of the drug and the skin barrier, thereby contributing to improved permeation [35,54]. To fully understand whether the ex vivo permeation observed is directly related to the effective drug release seen in vitro, it is essential to explore the mechanisms by which PG operates in both contexts. In vitro, PG enhances the release of C-PC by disrupting the hydrogel matrix, as discussed earlier. This increased release rate results in a higher concentration of C-PC available at the skin surface, which is a critical factor for initiating permeation through the stratum corneum. The elevated concentration gradient established by the more efficient release of C-PC is likely a significant driver for its enhanced ex vivo permeation [37]. However, the ex vivo environment introduces additional complexities that influence drug permeation. The stratum corneum serves as the primary barrier to drug penetration, and PG facilitates permeation by interacting with the lipid matrix within this layer. PG’s ability to fluidize lipids in the stratum corneum and increase its permeability is a key mechanism that must be considered. This interaction not only facilitates the entry of C-PC into the skin but also enhances its retention within the stratum corneum, as observed in ex vivo studies [35,37,54]. A study by Kis N. et al. demonstrated that PG increases lipid mobility within the stratum corneum, although the majority of the lipids remain in a solid phase even after treatment. PG significantly enhanced the mobility of amino acids in both keratin filaments and the natural moisturizing factor (NMF) free amino acids, acting similarly to NMF by replacing water in the stratum corneum [61]. In summary, the effective in vitro release of C-PC facilitated by PG is a critical precursor to its ex vivo permeation. The concentration of C-PC at the skin surface, modulated by PG in vitro, is pivotal for its subsequent diffusion into and through the stratum corneum. Without effective release, the availability of C-PC for permeation would be significantly reduced, leading to suboptimal therapeutic outcomes. Thus, the enhanced in vitro release of C-PC, driven by PG, correlates strongly with the observed ex vivo permeation results. PG not only promotes C-PC release from the hydrogel but also prepares the skin barrier for more efficient drug penetration. These dual roles of PG—in enhancing both release and permeation—create a synergistic effect that underlies the successful delivery of C-PC through the skin.

In conclusion, the effective in vitro release of C-PC in the presence of PG is indeed a critical factor that contributes to the observed ex vivo permeation. The mechanistic link between these two phenomena lies in PG’s ability to simultaneously enhance the solubility and diffusion of C-PC in vitro and modify the skin barrier ex vivo, thereby ensuring efficient drug delivery.

## 4. Materials and Methods

### 4.1. Materials 

C-PC was isolated from cyanobacteria biomass collected in Kaunas Lagoon (Kaunas, Lithuania). SharoSENSE Plus^TM^ 184 (maltol, didecyldimonium chloride), SharoSENSE^TM^ Plus 785 (maltol, sorbic acid, sodium benzoate), and Sharomix^TM^ 721 (dehydroacetic acid, benzyl alcohol, water) were received from SHARON Laboratories (Ashdod, Israel). Soligel^TM^ (a polysaccharide consisting of monomer units that include three glucose and three galactose molecules in the pyranose form, along with one glucuronic acid and a pyruvyl substituent) was received from Givaudan (Vernier, Switzerland). Carbopol^®^ Ultrez 21 (C10-30 alkyl acrylate crosspolymer) and Pemulen^TM^ (C10-30 alkyl acrylate crosspolymer) were received from The Lubrizol Corporation (Wickliffe, OH, USA). Poloxamer 407 (poly(oxyethylene)-poly(oxypropylene)-poly(oxyethylene) triblock copolymer), sodium alginate, hydroxyethyl cellulose, sodium carboxymethyl cellulose, hydroxy-propyl methylcellulose, methyl 2-hydroxyethyl cellulose, chitosan 80/1000, glycerol, and propylene glycol were purchased from Sigma-Aldrich (St. Louis, MO, USA). All reagents and solvents were of analytical grade. Purified deionized water was obtained using a Milli-Q^®^ water purification system (Millipore, Burlington, MA, USA). 

### 4.2. Collection of Cyanobacterial Biomass 

The biomass of cyanobacteria was collected with a plankton net (with a mesh size of 20 μm) from the Kaunas Lagoon (Kaunas, Lithuania) during a cyanobacteria bloom in September–October 2022. The collected biomass was frozen and stored at −20 °C in a freezer. In addition, phytoplankton samples were taken from the surface water layer (0.10–0.30 m) for microscopic analyses using a Ruttner water sampler to examine the bloom-forming species. Samples were preserved with a 4% (*v*/*v*) formaldehyde solution. Microscopic analysis revealed that species of the genus *Microcystis* prevailed in the samples collected in 2022 (up to 98%). 

### 4.3. C-PC Extraction and Purification 

C-PC extraction and purification were conducted using wild-collected cyanobacterial biomass following the method described by Khazi et al. (2018), with some modifications [62]. C-PC was extracted using one freeze–thaw cycle (freezing at −20 °C and thawing at 20 ± 2 °C). The supernatant for purity assessment was collected after centrifugation at 8000× *g* for 10 min and precipitated with a 50% saturated ammonium sulfate solution overnight at +4 °C. The ammonium sulfate-precipitated solution was centrifuged at 10,000× *g* for 10 min, and the precipitate was resuspended with 10 mM sodium phosphate buffer (pH 7.0). Desalting was performed via diafiltration using a membrane with a 10 kDa pore size. Gel filtration chromatography on a Sephadex G-25 column was used to purify the C-PC from the biomass with a dominant presence of *Microcystis* spp. The column was pre-equilibrated and eluted with 10 mM sodium phosphate buffer (PBS), pH 7.0, at a flow rate of 0.5 mL/min. The purified C-PC solution was rapidly frozen at −40 °C and then freeze-dried using a VaCo 2 freeze dryer (Zirbus Technology, Bad Grund, Harz, Germany) at a vacuum pressure of about 0.05 mbar for approximately 24–36 h. The freeze-dried C-PC powder was stored at −20 °C.

### 4.4. Purity Assessment of C-PC 

The purity of C-PC was determined spectrophotometrically, according to the following formula (Bennett, Bogodar, 1973) [63]:

C-PC purity = OD620/OD280,

where OD280 indicates the absorbance of total proteins and OD620 indicates the absorbance of C-PC. The experiment was repeated three times. 

### 4.5. Production of Experimental C-PC Hydrogels

The 1% (*w*/*w*) C-PC gels were prepared using various gelling agents, including chitosan (3% *w*/*w*), sodium alginate (3.5% *w*/*w*), Soligel^TM^ (1.5% *w*/*w*), HEC (5.5% *w*/*w*), NaCMC (5% *w*/*w*), HPMC (15% *w*/*w*), HEMC (3% *w*/*w*), Carbopol^®^ Ultrez 21 (0.8% *w*/*w*), Pemulen^TM^ (0.5% *w*/*w*), and poloxamer 407 (15% *w*/*w*). Lyophilized C-PC powder was dissolved in purified water. The appropriate amount of gelling agent was added and mixed using an IKAMAG^®^ C-MAG HS7 magnetic stirrer with a heated surface (IKA-Werke GmbH & Co. KG, Staufen, Germany). The mixture was stored at +4 °C until swelling occurred. For the preparation of the chitosan hydrogel, chitosan and purified water were heated to +30 °C. Acetic acid was added drop by drop until the chitosan dissolved and a transparent hydrogel formed. Lyophilized C-PC powder dissolved in purified water was homogeneously mixed with the prepared chitosan gel. For Carbopol^®^ Ultrez 21, the mixture was neutralized with an 18% sodium hydroxide solution to achieve the desired pH value (5.5–7.5) and stirred until a homogeneous hydrogel was obtained. In the production of the poloxamer 407 hydrogel, triethanolamine was used for neutralization, and the mixture was stirred until a homogeneous hydrogel formed. When producing hydrogels with glycerol, PG, and preservatives (SharoSENSE PlusTM 184, Sharosense 785, and Sharomix 721), these substances were dissolved in purified water together with lyophilized C-PC powder. C-PC hydrogels were stored at +4 °C for 48 h prior to spectrophotometric analysis.

### 4.6. Spectrophotometric Analysis

The amount of C-PC in the produced hydrogels was determined using the spectrophotometric method. The calibration curve (y=1.8673x−0.0043; $R^{2}=0.9998$) was produced using C-PC solutions in PBS of a 0.1–0.6 mg/mL concentration (n=11) with 3 replicates. The absorbance of the solutions was measured at a wavelength of 620 nm. 

The C-PC hydrogel was placed into PBS (pH 7.4) and stirred at 100 rpm for 30 min, then centrifuged at 10,000× *g* rpm for 10 min at 25 °C. In this way, the gel matrix was broken, resulting in a clear C-PC solution in the PBS. The amount of C-PC determined spectrophotometrically was compared with the amount initially added to formulate the hydrogel. The repeatability of each spectrophotometric experiment, during which the amount of C-PC in the hydrogel formulation was determined, was assessed over three trials. 

### 4.7. Microbiological Analysis

The microbiological examination of C-PC hydrogels was conducted at the Kaunas Department of the National Public Health Care Laboratory (Kaunas, Lithuania) to evaluate the microbiological contamination. The microorganisms tested were selected based on the Scientific Committee on Consumer Safety Notes of Guidance for the Testing of Cosmetic Ingredients and Their Safety Evaluation [53]. The microbiological methods used to examine the presence of *Candida albicans*, *Escherichia coli*, *Pseudomonas aeruginosa*, *Staphylococcus aureus*, and the total count of aerobic mesophilic bacteria were chosen according to international standards, as detailed in Table 3.

### 4.8. Determination of pH of Experimental C-PC Hydrogels 

The pH of the C-PC experimental formulations (*n* = 3) was assessed using a pH-Meter 766 Calimatic (Knick Elektronische Messgeräte GmbH & Co, Berlin, Germany).

### 4.9. In Vitro Study of C-PC Release from Experimental C-PC Hydrogels

In vitro release studies were carried out in triplicate (*n* = 3) using modified Franz-type diffusion cells [69,70,71]. An infinite dose of the donor phase (~1.00 g) was loaded into the cell with a polyvinylidene difluoride (PVDF) 0.22 μm dialysis membrane Durapore^®^ (Merck KGaA, Darmstadt, Germany). The diffusion area was 1.33 cm^2^. The experimental conditions did not limit the solubility and dissolution of C-PC in the PBS acceptor medium (the volume of the acceptor medium was 5 mL) during the diffusion process. The acceptor medium was mixed using an IKAMAG^®^ C-MAG HS7 magnetic stirrer with a heated surface (IKA-Werke GmbH & Co.KG, Staufen, Germany) and maintained at 32 ± 0.1 °C. Acceptor medium samples (1.0 mL) were taken after 0.5, 1, 2, 3, 4, 5, and 6 h. The amount of C-PC released was evaluated using the spectrophotometric method. The C-PC flux was expressed as the amount of C-PC released per cm^2^ per unit of time.

### 4.10. Ex Vivo Skin-Permeation Study of C-PC

Ex vivo skin-permeation studies of C-PC were performed using Bronaugh-type flow-through diffusion cells following the method described by Žilius M. et al. with some modifications [72]. The study was carried out using Caucasian women’s abdominal skin obtained from the Department of Plastic and Reconstructive Surgery, Hospital of the Lithuanian University of Health Sciences, after cosmetic surgery. The Kaunas Region Bioethical Committee has approved the use of human skin for transdermal penetration studies, with ethical approval code BE-2-42 (3 May 2023). The effective diffusion area in the cells was 0.64 cm^2^. The skin samples (*n* = 3 per formulation) were pre-equilibrated in a PBS solution at +25 °C for 12 h before the experiments. After equilibration, an infinite dose of the donor phase was applied to the skin cells, approximately 500 mg of hydrogel. The cells were sealed with Parafilm and covered with aluminum foil to protect them from light. The ex vivo skin-penetration tests were carried out for 24 h. At the end of the experiment, the skin specimens were gently washed with PBS solution and placed in a 4% (*v*/*v*) formalin solution for 3 h. Then, the skin samples were washed with a PBS solution and placed in a 30% (*v*/*v*) sucrose solution for 12 h.

The skin was embedded in a Shandon Cryomatrix compound (Thermo Fisher Scientific, Waltham, MA, USA) and frozen at −60 °C on a cooling stage. The frozen skin samples were sectioned at 20μm using a cryostat microtome (Microm HM 560, Leica, Wetzlar, Germany), then examined by confocal laser scanning microscopy (CLSM) (LSM 700, ZEISS, Oberkochen, Germany). C-PC was detected using a 639 nm diode laser. Using a Plan-Apochomat 20x objective with a numerical aperture (NA) of 0.55, images with a field size of 2048 μm × 2048 μm were generated. Transverse skin-section images were collected using ZEN 2010 software, and the z-stack method was applied to evaluate the accumulation of C-PC in the skin. C-PC was excited at 651 nm and detected at 670 nm. A Cy3 filter was utilized to isolate tissue autofluorescence and digitally assign the emission to a green color channel.

### 4.11. Statistical Analysis of Data

The research results were statistically calculated and analyzed using IBM SPSS Statistics version 29.0. (SPSS Inc., Chicago, IL, USA). The results were expressed as the mean and standard deviation. A paired-sample *t*-test was used to compare two dependent samples, while an independent samples *t*-test was used to compare two independent samples (*p* < 0.05). Bonferroni’s test was applied to the comparison of more than two independent samples (*p* < 0.05).

## 5. Conclusions

The study demonstrated that polysaccharide biopolymers, such as sodium alginate, hydroxyethyl cellulose, and SoligelTM, are effective at formulating C-PC hydrogels while preserving the stability of C-PC. The preservative SharoSENSETM Plus 785 exhibited excellent antimicrobial properties and compatibility with C-PC in the semisolid formulation. An in vitro release study indicated that, after 6 h, C-PC release from the sodium alginate hydrogel containing PG was approximately 10%, or 728.07 (±19.35) μg/cm^2^, which was significantly higher than the nearly 7%, or 531.44 (±26.81) μg/cm^2^, release from the hydrogel without PG (*p* < 0.05). Furthermore, an ex vivo qualitative skin-permeation study revealed that PG enhanced the penetration of C-PC into the outer layer of the skin, the stratum corneum. However, for C-PC to exert its effective antioxidant and anti-inflammatory effects on the skin, mere penetration into the stratum corneum may not be sufficient. Therefore, it is advisable to further develop technological formulations that can encapsulate C-PC molecules and improve drug delivery.

## Figures and Tables

**Table 1:** Results of microbiological evaluation of C-PC hydrogels.

Microorganisms	Hydrogel Samples		
	I C-PC Hydrogel	II C-PC Hydrogel	III C-PC Hydrogel
*Candida albicans*	Not detected	Not detected	Not detected
*Escherichia coli*	Not detected	Not detected	Not detected
*Pseudomonas aeruginosa*	Not detected	Not detected	Not detected
*Staphylococcus aureus*	Not detected	Not detected	Not detected
Number of aerobic mesophilic bacteria CFU */g	<1.0 × 10^1^	<1.0 × 10^1^	<1.0 × 10^1^

* The number of microorganisms or colony-forming units of a group of microorganisms in a certain unit of volume or mass.

**Table 2:** Composition and pH of experimental C-PC hydrogels.

	Excipient	C-PC Hydrogel without PG (C-PC Hydrogel) (%, *w*/*w*)	C-PC Hydrogel with PG (C-PC PG Hydrogel) (%, *w*/*w*)
**Composition of Experimental Hydrogels**	Purified water	90	75
	Sodium alginate	3.5	3.5
	C-PC	1	1
	Glycerol	5	5
	PG	-	15
	SharoSENSE^TM^ Plus 785	0.5	0.5
Determined pH		5.0 (0.03)	4.8 (0.01)

**Table 3:** Methods of microbiological testing of C-PC hydrogels.

Microorganisms	Method for Detecting Microorganisms
*Candida albicans*	LST EN ISO 18416:2016 except ISO 18416:2015/Amd1:2022 (N) [64]
*Escherichia coli*	LST EN ISO 21150:2016 except ISO 21150:2015/Amd1:2022 (N) [65]
*Pseudomonas aeruginosa*	LST EN ISO 22717:2016 except ISO 22717:2015/Amd1:2022 (N) [66]
*Staphylococcus aureus*	LST EN ISO 22718:2016 except ISO 22718:2015/Amd1:2022 (N) [67]
Number of aerobic mesophilic bacteria CFU */g	LST EN ISO 21149:2017 except ISO 21149:2017/Amd1:2022 (N) [68]

* The number of microorganisms or colony-forming units of a group of microorganisms in a certain unit of volume or mass.

## Data Availability

The data supporting the findings of this study are available from the corresponding author [D.G.] upon reasonable request.
